# Baseline Performance and the 72‐Hour Performance Window Following Plyometric Withdrawal in Collegiate Sprinters

**DOI:** 10.1002/ejsc.70277

**Published:** 2026-10-20

**Authors:** Hao Du, Jingkun Zhou, Yuxiang Liu, Lei Yang, Yonggang Luo, Shuai Yang

**Affiliations:** ^1^ School of Humanities and Physical Education Hebei Oriental College Langfang Hebei China; ^2^ School of Arts and Physical Education Huanghe Science and Technology College Zhengzhou Henan China; ^3^ School of Physical Education Langfang Normal University Langfang Hebei China; ^4^ Physical Education Department Qinghai University Xining Qinghai China; ^5^ Emilio Aguinaldo College Manila Philippines

**Keywords:** countermovement jump, microcycle, plyometric training, sprint performance, training withdrawal

## Abstract

Plyometric studies emphasise pre–post change, but coaches need to know what remains after the block ends. This single‐cohort study followed 26 collegiate sprinters through 12 weeks of Monday plyometric training and 4 weeks of withdrawal, without a parallel control group. During intervention, 30 m sprint time and arm‐assisted countermovement jump (CMJ) height were measured at 0, 24, 48, 72, and 96 h; 0 and 72 h were retained during withdrawal. Mixed models showed large week effects for sprint, F(1, 1534) = 2272.01, partial *η* ^2^ = 0.597, 95% CI [0.570, 0.622], and CMJ, F(1, 1534) = 2537.82, partial *η* ^2^ = 0.623, 95% CI [0.598, 0.647]. Time‐point effects were evident, but week × time‐point interactions were not. Relative to 0 h, sprint was 0.0098 s slower at 24 h and 0.0057 s faster at 72 h; CMJ was 0.311 cm lower at 24 h and 0.245 cm higher at 72 h (all Tukey‐adjusted *p* < 0.001). Linear, quadratic, and autoregressive integrated moving average (ARIMA) models were compared by rolling one‐step error. ARIMA had the lowest rolling errors and provided exploratory, model‐based references for weeks 13–16. During withdrawal, 1/4 sprint and 0/4 CMJ 0 h observations fell within the 95% prediction interval; mean absolute deviations were 0.0233 s and 0.794 cm. Three of four 72 − 0 h observations were within the interval for both outcomes. The 72 − 0 h series more often fell within their model‐based intervals than the 0 h series, indicating different short‐term patterns.

## Highlights

- Sprint and arm‐assisted CMJ improved across 12 weeks, while the within‐week response profile remained broadly stable.
- Performance was less favourable at 24 h and more favourable at 72 h after the structured Monday plyometric session.
- After withdrawal, the 72 − 0 h window remained closer to the short‐horizon model‐based references than the weekly pre‐session level.

## 1 Introduction

Plyometric training is widely used in sprint preparation because it targets rapid force production and stretch–shortening‐cycle function during brief ground contacts. Reviews and meta‐analyses have reported the most consistent gains in jump performance, with smaller but practically relevant improvements in short sprint performance when training is progressive and sustained (Markovic 2007; Sáez de Villarreal et al. 2012; Oxfeldt et al. 2019; Kons et al. 2023; Zheng et al. 2025). Responses nevertheless vary with training status, the performance task, programme content, and the other work completed within the microcycle. In sprint programmes, this matters because plyometric exercises are usually added to acceleration, maximal‐velocity, technical, and speed‐endurance training rather than delivered in isolation.

Less is known about performance after a structured plyometric block. Most interventions end with a post‐test, showing change during the programme but not whether it remains evident in the next training phase. Detraining research suggests that strength, speed, and power do not decline uniformly, particularly when task‐specific training continues after one component is reduced or removed (Mujika and Padilla 2000; Pereira et al. 2020; Nieto‐Acevedo et al. 2025). Post‐block assessment should therefore consider both the performance level carried forward and its expression across the training week.

Within‐week timing also affects interpretation. Repeated maximal jumping and sprinting can impair next‐day performance, with recovery extending 48–72 h depending on the task and training dose (Thomas et al. 2018; Doma et al. 2021). The 72 h assessment was selected before the withdrawal analysis because the early decrement could subside, it preceded the Friday sprint class, and it could be repeated at the same point in each microcycle. The 96 h assessment examined whether a favourable response persisted beyond 72 h; the pre‐specified withdrawal series was 72 − 0 h.

Weekly 0 h performance represented the level immediately before Monday plyometrics, whereas 72 − 0 h described relative within‐week change. The 0 h series is termed baseline for brevity, but it was neither untrained nor physiologically isolated because regular sprint training, including the Friday class, continued. Candidate models were compared by rolling one‐step prediction error to provide exploratory, model‐based references for weeks 13–16 rather than by in‐sample fit, thereby evaluating each prediction against a value not used to estimate that step (Tashman 2000; Hyndman and Khandakar 2008).

The study examined whether these two features followed the same course. The first question was whether pre‐session sprint and CMJ performance improved over 12 weeks while the 0–96 h profile remained broadly similar from week to week. The second was whether, after removal of the structured Monday plyometric session, the 0 h series and the pre‐specified 72 − 0 h series remained similarly close to their model‐based forecast references. We expected favourable week‐to‐week changes in sprint and CMJ performance, less favourable values at 24 h, more favourable values at 72 h, and little evidence of a week × time‐point interaction. During withdrawal, we expected the proximity to the model‐based forecast reference to differ by task and not necessarily to be the same for the absolute 0 h level and the relative 72 h window.

## 2 Methods

### 2.1 Ethics Statement

At the time of data collection, the host institution did not have a formal institutional research ethics committee, and no institutional ethics approval number was available. The study was conducted within the athletes' scheduled training environment under the supervision of departmental staff, sprint coaches, and a rehabilitation teacher. Before participation, each athlete received an explanation of the procedures, provided written informed consent, and was informed that withdrawal was possible at any time without penalty. The study procedures followed the principles of the Declaration of Helsinki. This paragraph describes the institutional arrangements in place when the study was conducted and should not be interpreted as retrospective ethical approval.

### 2.2 Participants

The cohort comprised 26 male third‐year collegiate sprinters from the host institution (age 20.69 ± 0.88 years, height 176.08 ± 4.06 cm, body mass 74.25 ± 4.03 kg). Their main events were the 100 m (*n* = 15), 200 m (*n* = 9), and 400 m (*n* = 2). Nineteen athletes competed at the university level, six at the city level, and one at the provincial level. Mean general training experience was 5.92 ± 0.39 years, including 2.98 ± 0.48 years of sprint‐specific training. Personal‐best performances were 11.37 ± 0.27 s for the 100 m group, 23.57 ± 1.05 s for the 200 m group, and 54.95 ± 1.94 s for the 400 m group.

Eligibility required membership of the third‐year sprint squad, at least 5 years of general and more than 2 years of sprint‐specific training, availability for the full 16 weeks, no lower‐limb injury restricting training or testing, and no current medication expected to affect performance. All 26 eligible athletes were enroled before data collection; none were added later, and each completed a health screening.

The sample therefore represented the complete eligible and available squad rather than a number derived from a two‐group power calculation. Once the final model structure had been established, a simulation‐based sensitivity analysis was used to characterise the effects that could be detected under the realised repeated‐measures design. The analysis used *α* = 0.05 and 2000 simulations for each pre‐specified effect scenario. It was not treated as a prospective recruitment calculation or as a post hoc power based on the observed effects.

### 2.3 Design and Weekly Training Context

A single‐cohort longitudinal design was used. During weeks 1–12, athletes completed a structured plyometric session each Monday from 13:30 to 15:00. The independent Monday session was then removed for a 4 week observation phase.

Testing occurred from 12:50 to 13:30 immediately before Monday's training (0 h) and at 24, 48, 72, and 96 h. The regular Friday sprint class ran from 16:00 to 17:30 after the 96 *h* test; it contributed to exposure before the next Monday's 0 *h* test but not the same week's 96 h result. The complete Friday sprint schedule across weeks 1–16 is provided in Supporting Information S1: Table S5.

During weeks 13–16, testing continued at 0 and 72 h, but the structured Monday plyometric session was no longer performed. The Friday sprint class continued together with the standard warm‐up and a small amount of auxiliary jumping embedded in normal sprint teaching. No replacement plyometric block, separate resistance session, or additional explosive‐training programme was introduced. Throughout this paper, withdrawal therefore refers specifically to the removal of the independent Monday plyometric session, not to the cessation of sprint training or all jump exposure.

### 2.4 Plyometric Programme

The programme was delivered in the same indoor gymnasium by members of the research team and specialist teaching staff. It consisted of three 4 week phases, with all athletes using the same ordinary running shoes across the Monday sessions.

Each exercise comprised 3 sets with 1 min between sets. Between‐exercise recovery was 3 min in the first 2 weeks and 4 min in the final 2 weeks of each phase. Unilateral sets used the left leg and right leg, then preferred force‐production leg. Supporting Information S1: Table S1 provides the complete prescription, repetitions, and weekly contacts.

Weeks 1–4 comprised unilateral and bilateral vertical, lateral, and zig‐zag hurdle jumps. Weekly contacts increased from 144 to 216. Weeks 5–8 introduced box jumps, depth‐jump‐to‐unilateral‐hurdle combinations, lateral push‐off‐to‐box jumps, and 2 medicine‐ball throwing exercises. Jump contacts increased from 78 to 132; medicine‐ball repetitions were recorded separately and were not included in the contact totals. Weeks 9–12 included depth‐jump‐to‐box, unilateral tuck‐jump, half‐squat‐to‐box, cyclical split‐squat‐jump, and split‐scissor‐jump sequences, with weekly contacts increasing from 132 to 198. Progression was achieved through changes in exercise complexity and prescribed repetitions.

Exact hurdle, box, and drop heights, medicine‐ball mass, and the gym‐surface specification were absent from the contemporaneous records and were not reconstructed.

### 2.5 Standardised Warm‐Up and Pre‐Test Controls

The same 10 min warm‐up was completed before each performance assessment, Monday plyometric session, and Friday sprint class. The first 4 min consisted of controlled movements of the head, shoulders, trunk, hips, lower limbs, and ankles. The following 6 min combined low‐amplitude ankle jumps, A‐jumps, alternate bounds, high‐knee running, low‐amplitude single‐leg exchanges, short‐stride running, and progressive acceleration. The final 40–50 m acceleration reached approximately 85%–90% effort but did not include an all‐out sprint. The complete sequence is presented in Supporting Information S1: Table S2.

Athletes were instructed to avoid caffeine and alcohol throughout the study, maintain their usual diet, and follow the existing team training schedule without adding study‐related training. Water was available during recovery periods. For a given task, each athlete used the same footwear throughout the 16 weeks: ordinary running shoes for CMJ testing and the gym‐based plyometric session, and the same pair of sprint spikes for the 30 m test. Footwear was inspected before testing for non‐compliant mechanical assistance.

### 2.6 Performance Assessments

#### 2.6.1 Arm‐Assisted Countermovement Jump

CMJ was assessed beside the indoor track against the same flat white wall as an arm‐assisted reach‐difference jump, not with a force platform, jump mat, or flight‐time calculation.

While wearing the designated running shoes, each athlete first stood with one arm fully extended overhead. A small marker was attached to the finger, with its upper edge aligned with the highest fingertip position, to establish standing reach. The athlete then performed a vertical jump using a self‐selected countermovement depth and arm swing and placed the marker at the highest point reached on the wall. A Mijia MJJGCJY001QW laser distance metre (Xiaomi, China) was used to measure the vertical distance from the same floor reference to the upper edge of the marker. Jump height was calculated by subtracting standing reach from jumping reach. Knee angle and countermovement depth were not instrumented.

Three trials were completed at each time point, with a minimum of 3 min between attempts. When 3 min had not elapsed, the next trial was postponed until the required recovery period had been completed. The arithmetic mean of the three trials was used in the analysis. A specialist assistant teacher supervised testing, and the room, wall, device, test order, and calculation procedure remained unchanged throughout the study.

#### 2.6.2 30 m Sprint

After completing the CMJ assessment, athletes moved directly to the adjacent indoor track. Sprint time was recorded using a CC01 single‐point infrared electronic timing system. Two opposing units at the start and two at the finish created the respective infrared beams. Timing began when the athlete broke the start beam and stopped when the finish beam was crossed. Only one athlete was tested at a time.

During week 1, each athlete selected a preferred lane and continued to use that lane for all subsequent assessments. Two 30 m trials were completed at each time point in the athlete's designated sprint spikes. The minimum recovery period between trials was 10 min. When an athlete returned to the testing position before 10 min had elapsed, the second attempt was delayed until the full interval had been completed.

The arithmetic mean of the two electronic times was analysed. Individual trials were not retained after transfer, preventing retrospective trial‐level reliability estimates.

### 2.7 Training Monitoring and Safety

Previous‐night sleep duration and lower‐limb soreness, rated from 0 to 10, were recorded to describe the weekly recovery context. The rating of perceived exertion for the Monday session was collected between 15:20 and 15:30, and the training load was calculated as session‐RPE × 90 min (Haddad et al. 2017). These measures were retained as descriptive information. They were not entered as primary covariates because the analysis focussed on repeated performance trajectories rather than on modelling day‐to‐day variation from multiple explanatory variables.

All 26 athletes completed the 12 Monday sessions and every planned assessment. Two athletes reported brief dizziness and one brief nausea; a rehabilitation teacher examined each athlete. Symptoms resolved after 3–4 min of rest without medication or referral, and all three continued trainings and testing that day. No serious adverse events, protocol modifications, or withdrawals occurred. Adherence, safety, and withdrawal‐phase exposure are summarised in Supporting Information S1: Table S3.

### 2.8 Derived Outcomes

Four weekly group‐mean series were defined for the withdrawal analysis:

1. 30 m sprint time at 0 h;
2. CMJ height at 0 h;
3. Sprint 72 − 0 h difference; and
4. CMJ 72 − 0 h difference.

For concision, the two 0 h outcomes are referred to as the baseline series. In this context, baseline denotes the weekly pre‐session performance level and not an untrained or physiologically isolated condition. A negative sprint difference indicated that the 72 h time was faster than the corresponding 0 h time, whereas a positive CMJ difference indicated a higher jump at 72 h. The 72 h contrast had been specified before the withdrawal values were analysed.

### 2.9 Statistical Analysis

Analyses were conducted in *R* version 4.4.3. Descriptive results are presented as mean ± SD.

Separate linear mixed models were fitted to 30 m sprint time and CMJ height over weeks 1–12. Week was entered as a continuous variable and centred at week 6.5. The time point was modelled as a 5‐level factor—0, 24, 48, 72, and 96 h—using sum‐to‐0 contrasts. Each model contained the fixed effects of week, time point, and their interaction, with a participant random intercept to account for repeated observations.

Omnibus effects were tested using Type III *F* tests with Satterthwaite denominator degrees of freedom (Kuznetsova et al. 2017). Estimated marginal means at the centred week were used for the pre‐specified comparisons between 0 h and each subsequent time point. Tukey adjustment was applied across the full set of pairwise comparisons. Omnibus effects are reported as partial η^2^ with two‐sided 95% confidence intervals. Pairwise estimates were additionally standardised using the model residual SD. Because this standardisation is not equivalent to the conventional independent‐group Cohen's d, the raw‐unit estimates and Tukey‐adjusted confidence intervals were treated as the primary comparison measures (Lakens 2013).

Residual plots were inspected alongside formal model checks. Shapiro–Wilk and Breusch–Pagan tests were reported, and participant‐specific lag‐1 residual correlations were calculated. The diagnostics indicated modest departures from normality and homoscedasticity, together with within‐participant serial dependence. Three sensitivity analyses were therefore conducted: participant‐clustered CR2 standard errors, continuous‐time CAR(1) models with time expressed in days, and models with participant‐specific week slopes. These analyses were used to examine whether the week effect, the principal 24 and 72 h directions, and the week × time‐point result were sensitive to alternative error or random‐effect structures. They did not replace the Tukey‐adjusted pairwise comparisons (Pustejovsky and Tipton 2018).

For each withdrawal series, linear, quadratic, and autoregressive integrated moving average (ARIMA) candidates were evaluated using expanding‐window, rolling one‐step forecasts. Mean absolute error (MAE) and root mean square error (RMSE) were calculated from the held‐out predictions. The model with the lowest rolling errors was then fitted to the 12 intervention‐week values to generate exploratory, model‐dependent forecasts for weeks 13–16, together with 80% and 95% prediction intervals (PIs).

ARIMA orders, augmented Dickey–Fuller and KPSS stationarity checks, and lag‐4 Ljung–Box residual tests were recorded. Because each series contained only 12 intervention values, the stationarity and residual checks were treated as supporting diagnostics rather than definitive model validation (Hyndman and Khandakar 2008; Hyndman and Athanasopoulos 2021).

Withdrawal was summarised by 95% PI coverage and the mean absolute deviation (MAD) between the four observed and forecast values. PI inclusion indicated proximity to the model‐based reference, not a null‐hypothesis test or physiological retention threshold.

There were no missing outcome values. The simulation‐based sensitivity analysis used 2000 datasets for each scenario and the final mixed‐model structure. Selected simulation‐based sensitivity scenarios are reported in Supporting Information S1: Table S4.

## 3 Results

### 3.1 Data Completeness and Training Context

Intervention data comprised 312 participant‐weeks and 312 Monday session‐RPE records; withdrawal contributed 104 participant‐weeks. All 26 athletes completed 12 Monday sessions and 68 testing occasions (1768 participant‐assessments). No primary outcomes were missing, and there were no withdrawals, unplanned assessments, or protocol substitutions. Mean session‐RPE ranged from 3.65 ± 1.09 to 7.42 ± 1.06, equivalent to 328.85–668.08 arbitrary units.

### 3.2 Intervention‐Period Trajectories

Table 1 shows the weekly 30 m sprint times across the 12 week intervention.

**TABLE 1:** Weekly 30 m sprint time during the 12 week intervention (*n* = 26; mean ± SD, s).

Week	0 h	24 h	48 h	72 h	96 h
1	4.400 ± 0.025	4.409 ± 0.026	4.403 ± 0.024	4.395 ± 0.026	4.398 ± 0.026
2	4.395 ± 0.024	4.408 ± 0.025	4.396 ± 0.024	4.389 ± 0.026	4.393 ± 0.024
3	4.393 ± 0.025	4.400 ± 0.028	4.398 ± 0.027	4.387 ± 0.028	4.391 ± 0.027
4	4.389 ± 0.026	4.397 ± 0.025	4.393 ± 0.025	4.383 ± 0.025	4.387 ± 0.026
5	4.379 ± 0.030	4.393 ± 0.030	4.383 ± 0.031	4.375 ± 0.031	4.377 ± 0.029
6	4.372 ± 0.034	4.384 ± 0.037	4.376 ± 0.034	4.366 ± 0.035	4.371 ± 0.035
7	4.365 ± 0.037	4.374 ± 0.036	4.369 ± 0.038	4.358 ± 0.038	4.363 ± 0.037
8	4.354 ± 0.036	4.365 ± 0.036	4.357 ± 0.037	4.347 ± 0.038	4.353 ± 0.035
9	4.352 ± 0.037	4.358 ± 0.037	4.354 ± 0.039	4.348 ± 0.038	4.350 ± 0.036
10	4.349 ± 0.036	4.357 ± 0.040	4.353 ± 0.036	4.345 ± 0.037	4.347 ± 0.037
11	4.346 ± 0.037	4.356 ± 0.035	4.349 ± 0.037	4.338 ± 0.037	4.346 ± 0.038
12	4.345 ± 0.037	4.355 ± 0.040	4.347 ± 0.039	4.341 ± 0.037	4.342 ± 0.037

*Note:* Lower values indicate faster sprint performance. Time points are expressed relative to the Monday structured plyometric session; 0 h was assessed immediately before the session.

Table 2 shows the weekly arm‐assisted CMJ heights across the same intervention period.

**TABLE 2:** Weekly arm‐assisted countermovement jump height during the 12 week intervention (*n* = 26; mean ± SD, cm).

Week	0 h	24 h	48 h	72 h	96 h
1	44.09 ± 1.74	43.78 ± 1.82	43.97 ± 1.71	44.44 ± 1.87	44.22 ± 1.73
2	44.27 ± 1.67	43.98 ± 1.62	44.17 ± 1.71	44.39 ± 1.59	44.33 ± 1.71
3	44.34 ± 1.70	44.01 ± 1.75	44.26 ± 1.72	44.53 ± 1.76	44.40 ± 1.71
4	44.46 ± 1.84	44.26 ± 1.82	44.37 ± 1.78	44.68 ± 1.97	44.57 ± 1.80
5	44.69 ± 1.80	44.31 ± 1.82	44.52 ± 1.80	44.99 ± 1.79	44.78 ± 1.83
6	44.87 ± 1.80	44.48 ± 1.89	44.80 ± 1.80	45.19 ± 1.75	44.90 ± 1.75
7	45.00 ± 1.72	44.64 ± 1.71	44.92 ± 1.74	45.21 ± 1.80	45.15 ± 1.73
8	45.14 ± 1.75	44.87 ± 1.87	45.05 ± 1.74	45.46 ± 1.72	45.24 ± 1.83
9	45.23 ± 1.72	45.01 ± 1.70	45.18 ± 1.69	45.46 ± 1.68	45.32 ± 1.75
10	45.32 ± 1.71	44.98 ± 1.90	45.24 ± 1.66	45.49 ± 1.75	45.41 ± 1.77
11	45.42 ± 1.74	45.05 ± 1.79	45.26 ± 1.75	45.63 ± 1.65	45.53 ± 1.77
12	45.48 ± 1.75	45.20 ± 1.86	45.39 ± 1.77	45.78 ± 1.76	45.50 ± 1.68

*Note:* Higher values indicate better performance. Jump height was obtained using an arm‐assisted reach‐difference procedure.

Abbreviation: CMJ, countermovement jump.

Across 12 weeks, mean 0 h sprint time decreased from 4.400 to 4.345 s and CMJ increased from 44.09 to 45.48 cm. Sprint was generally slowest at 24 h, near 0 h by 48 h, and fastest at 72 h. CMJ was lowest at 24 h, slightly below 0 h at 48 h, highest at 72 h, and often remained above 0 h at 96 h. The corresponding weekly descriptive values and trajectories are shown in Tables 1 and 2 and Figures 1 and 2.

**FIGURE 1:**
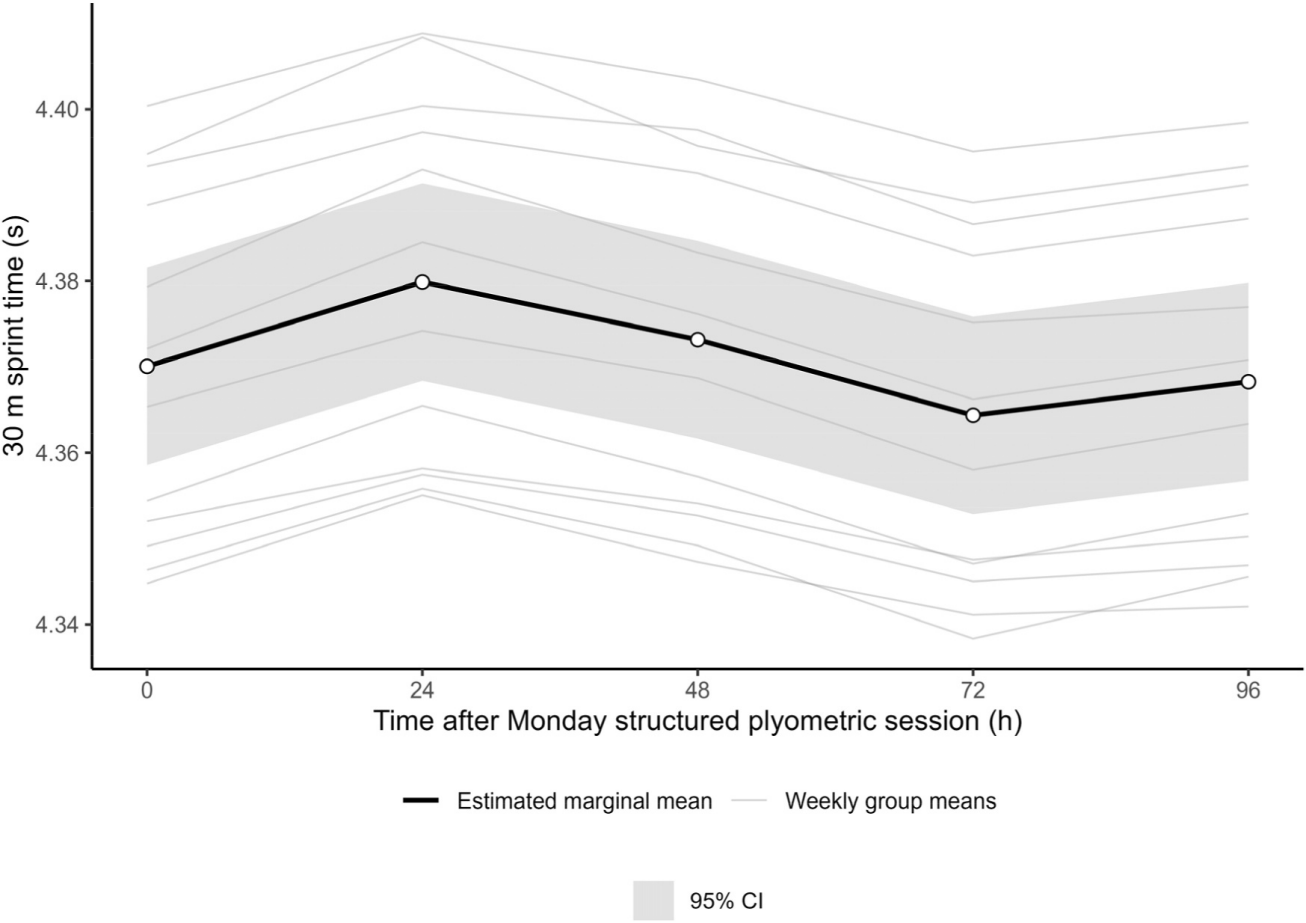
Weekly and model‐estimated within‐week trajectories for 30 m sprint time during the 12 week intervention. Thin lines represent weekly means. The thicker line represents the estimated marginal mean at the centred week, and the shaded region is its 95% confidence interval. Lower values indicate faster performance.

**FIGURE 2:**
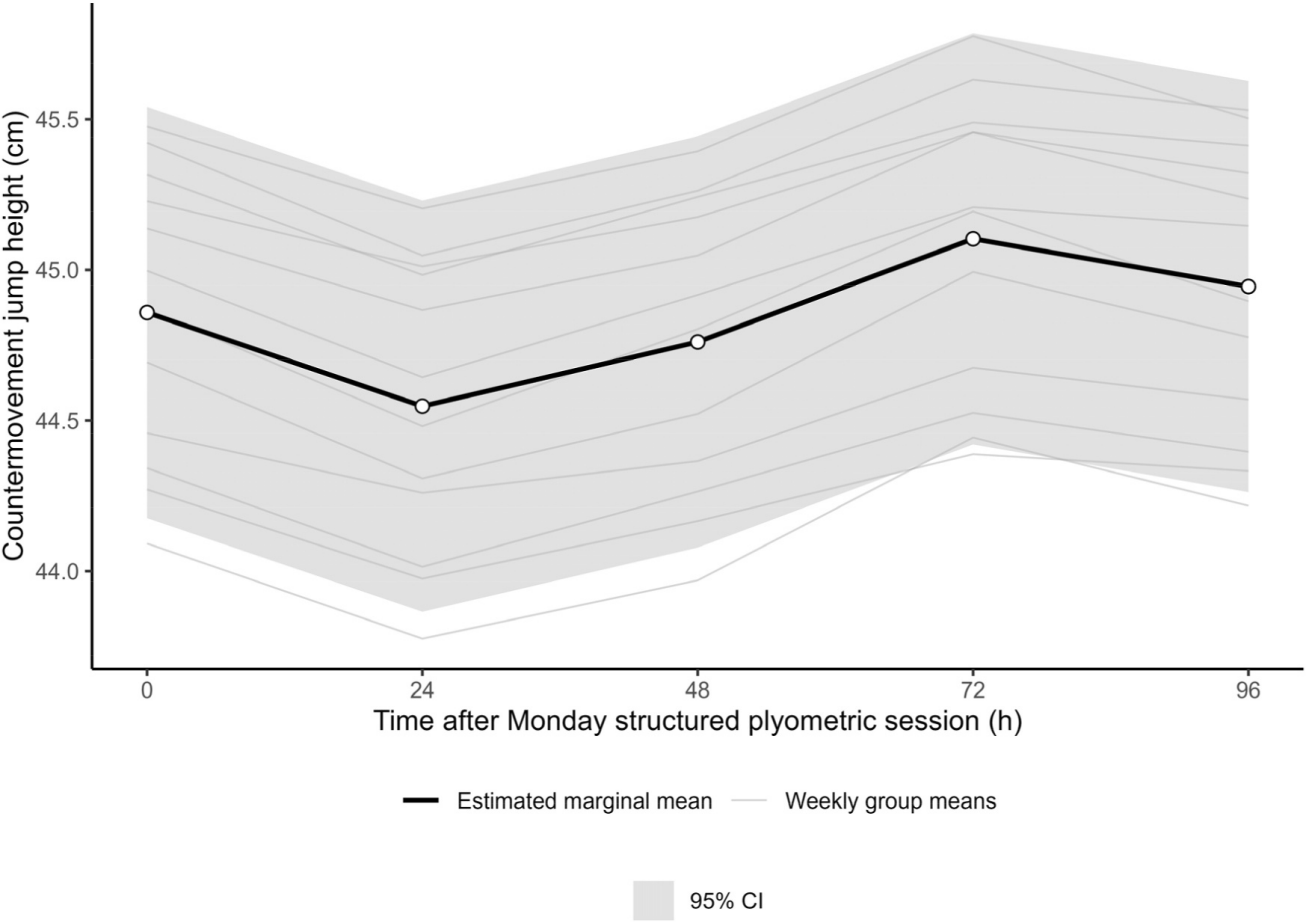
Weekly and model‐estimated within‐week trajectories for arm‐assisted countermovement jump height during the 12 week intervention. Thin lines represent weekly means. The thicker line represents the estimated marginal mean at the centred week, and the shaded region is its 95% confidence interval. Higher values indicate better performance.

### 3.3 Mixed‐Model Estimates

Table 3 shows the mixed‐model omnibus tests and pairwise comparisons for arm‐assisted CMJ height.

**TABLE 3:** Mixed‐model omnibus tests and comparisons with 0 h for arm‐assisted countermovement jump height.

Effect	Num. df	Den. df	*F*	*p*	Partial η^2^	95% CI
Week	1	1534	2537.819	< 0.001	0.623	[0.598, 0.647]
Time point	4	1534	105.164	< 0.001	0.215	[0.180, 0.249]
Week × time point	4	1534	0.023	0.999	< 0.001	[0, 0]

**Contrast (cm)**	**Estimate**	**SE**	**Tukey 95% CI**		**Tukey‐adjusted p**	
0 − 24 h	0.310929	0.028623	[0.232759, 0.389100]		< 0.001	
0 − 48 h	0.097788	0.028623	[0.019618, 0.175959]		0.0059	
0 − 72 h	−0.244615	0.028623	[−0.322786, −0.166445]		< 0.001	
0 − 96 h	−0.086314	0.028623	[−0.164484, −0.008144]		0.0219	

*Note:* Contrasts are expressed as 0 h minus the stated time point. A negative estimate indicates that CMJ height was greater at the later time point. Week was centred at 6.5. Omnibus effects were tested using Type III *F* tests with Satterthwaite denominator degrees of freedom. Pairwise confidence intervals and *p* values were Tukey‐adjusted.

Week and time point were associated with 30 m sprint performance, but no week × time‐point interaction was detected. At the centred week, sprint time was 0.00981 s slower at 24 h than at 0 h, with an adjusted 95% CI of 0.00625–0.01338 s slower. At 72 h, it was 0.00571 s faster than at 0 h, with an adjusted 95% CI of 0.00215–0.00927 s faster. The 48 and 96 h contrasts were smaller, and both confidence intervals included zero.

The CMJ model also showed week and time‐point effects without a detectable interaction. Relative to 0 h, CMJ height was 0.31093 cm lower at 24 h and 0.09779 cm lower at 48 h. It was 0.24462 cm higher at 72 h and 0.08631 cm higher at 96 h. Thus, the early decrement was still present at 48 h for CMJ, while its favourable response extended to 96 h. Neither outcome showed evidence that the within‐week profile changed systematically over the 12 week period. The omnibus tests and pairwise comparisons for both outcomes are reported in Tables 3 and 4.

### 3.4 Model Checks and Sensitivity Analyses

Diagnostics indicated departures from several assumptions of the random‐intercept models. Sprint residuals departed modestly from normality according to the Shapiro–Wilk test (*p* = 0.00041), whereas the CMJ residuals did not (*p* = 0.350). Breusch–Pagan tests indicated heteroscedasticity for both sprint (*p* = 0.00196) and CMJ (*p* = 0.00833). Mean participant‐specific lag‐1 residual correlations were 0.741 for sprint and 0.319 for CMJ. In the continuous‐time models, the estimated CAR(1) correlations were *ρ* = 0.865 per day for sprint and *ρ* = 0.334 per day for CMJ.

The participant‐clustered CR2, CAR(1), and non‐singular random‐slope analyses retained the direction of the week effect, the principal 24 and 72 h contrasts, and the absence of a detectable week × time‐point interaction. The unadjusted 48 and 96 h sprint coefficients were more sensitive to the assumed error structure. Interpretation of those comparisons therefore remained based on the pre‐specified Tukey‐adjusted contrasts in Table 4.

**TABLE 4:** Mixed‐model omnibus tests and comparisons with 0 h for 30 m sprint time.

Effect	Num. df	Den. df	*F*	*p*	Partial η^2^	95% CI
Week	1	1534	2272.008	< 0.001	0.597	[0.570, 0.622]
Time point	4	1534	39.954	< 0.001	0.094	[0.067, 0.121]
Week × time point	4	1534	0.042	0.997	< 0.001	[0, 0]

**Contrast (s)**	**Estimate**	**SE**	**Tukey 95% CI**		**Tukey‐adjusted p**	
0 − 24 h	−0.009814	0.001304	[−0.013376, −0.006252]		< 0.001	
0 − 48 h	−0.003099	0.001304	[−0.006661, 0.000462]		0.1223	
0 − 72 h	0.005708	0.001304	[0.002147, 0.009270]		< 0.001	
0 − 96 h	0.001801	0.001304	[−0.001760, 0.005363]		0.6399	

*Note:* Contrasts are expressed as 0 h minus the stated time point. A positive estimate indicates that sprint time was faster at the later time point. Week was centred at 6.5. Omnibus effects were tested using Type III *F* tests with Satterthwaite denominator degrees of freedom. Pairwise confidence intervals and *p* values were Tukey‐adjusted.

Under the simulation scenarios, the estimated power was 96.0% (95% CI 95.0%–96.8%) for a sprint slope of −0.001 s·week^−1^ and 86.9% (95% CI 85.3%–88.3%) for a −0.004 s sprint contrast at 72 h. For CMJ, the estimated power was 99.95% (95% CI 99.7%–100.0%) for a slope of 0.03 cm·week^−1^ and 94.4% (95% CI 93.3%–95.3%) for a 0.10 cm contrast at 72 h. These calculations refer to the stated assumed effects under the realised design and were not used to redefine the cohort as prospectively powered.

### 3.5 Forecast Selection and Withdrawal Comparison

ARIMA produced the lowest rolling MAE and RMSE for each of the four series. ARIMA(0,1,0) models with drift were selected for the sprint and CMJ 0 h series. ARIMA(0,0,0) models with a non‐zero mean were selected for both 72 − 0 h series. Candidate‐model forecast errors are summarised in Table 5.

**TABLE 5:** Rolling one‐step forecast errors for the candidate models.

Series	Model	MAE	RMSE
30 m sprint, 0 h	ARIMA	0.003525	0.003564
30 m sprint, 0 h	Linear	0.004446	0.005402
30 m sprint, 0 h	Quadratic	0.008167	0.008194
CMJ, 0 h	ARIMA	0.0556	0.0583
CMJ, 0 h	Linear	0.1043	0.1091
CMJ, 0 h	Quadratic	0.0785	0.0818
30 m sprint, 72 − 0 h	ARIMA	0.001957	0.001985
30 m sprint, 72 − 0 h	Linear	0.002436	0.002450
30 m sprint, 72 − 0 h	Quadratic	0.002985	0.003156
CMJ, 72 h − 0 h	ARIMA	0.0489	0.0533
CMJ, 72 h − 0 h	Linear	0.0666	0.0714
CMJ, 72 h − 0 h	Quadratic	0.0967	0.1099

*Note:* Sprint errors are reported in seconds and CMJ errors in centimetres. Errors were calculated from held‐out observations using expanding‐window one‐step forecasts.

Abbreviations: ARIMA, autoregressive integrated moving average; MAE, mean absolute error; RMSE, root mean square error.

Lag‐4 Ljung–Box tests did not indicate residual autocorrelation for sprint 0 h (*p* = 0.153), CMJ 0 h (*p* = 0.775), the sprint difference series (*p* = 0.371), or the CMJ difference series (*p* = 0.737). ADF and KPSS results were mixed for some series. Given that each intervention series contained only 12 observations, these stationarity checks were not treated as conclusive. The exploratory weeks 13–16 projections are reported in Table 6.

**TABLE 6:** Exploratory weeks 13–16 forecasts from intervention‐period models.

Series	Week	Forecast	80% PI	95% PI
30 m sprint, 0 h	13	4.339713	4.335191 to 4.344236	4.332797 to 4.346630
30 m sprint, 0 h	14	4.334657	4.328261 to 4.341053	4.324876 to 4.344439
30 m sprint, 0 h	15	4.329601	4.321768 to 4.337435	4.317621 to 4.341581
30 m sprint, 0 h	16	4.324545	4.315500 to 4.333591	4.310712 to 4.338379
CMJ, 0 h	13	45.602	45.530 to 45.673	45.493 to 45.710
CMJ, 0 h	14	45.727	45.627 to 45.828	45.574 to 45.881
CMJ, 0 h	15	45.853	45.730 to 45.976	45.665 to 46.042
CMJ, 0 h	16	45.979	45.837 to 46.121	45.761 to 46.197
30 m sprint, 72 − 0 h	13	−0.005708	−0.007537 to −0.003880	−0.008504 to −0.002912
30 m sprint, 72 − 0 h	14	−0.005708	−0.007537 to −0.003880	−0.008504 to −0.002912
30 m sprint, 72 − 0 h	15	−0.005708	−0.007537 to −0.003880	−0.008504 to −0.002912
30 m sprint, 72 − 0 h	16	−0.005708	−0.007537 to −0.003880	−0.008504 to −0.002912
CMJ, 72 h − 0 h	13	0.245	0.151 to 0.338	0.102 to 0.387
CMJ, 72 h − 0 h	14	0.245	0.151 to 0.338	0.102 to 0.387
CMJ, 72 h − 0 h	15	0.245	0.151 to 0.338	0.102 to 0.387
CMJ, 72 h − 0 h	16	0.245	0.151 to 0.338	0.102 to 0.387

*Note:* Sprint values are reported in seconds and CMJ values in centimetres. These forecasts are exploratory, model‐dependent extensions of the week 1–12 series, not observed outcomes under continued training or data from a control group.

Abbreviation: PI, prediction interval.

The 0 h series diverged differently: one sprint observation was within the 95% PI (MAD 0.0233 s), whereas no CMJ observations were within the interval (MAD 0.794 cm). The corresponding observed and forecast pre‐session trajectories are shown in Figures 3 and 4.

**FIGURE 3:**
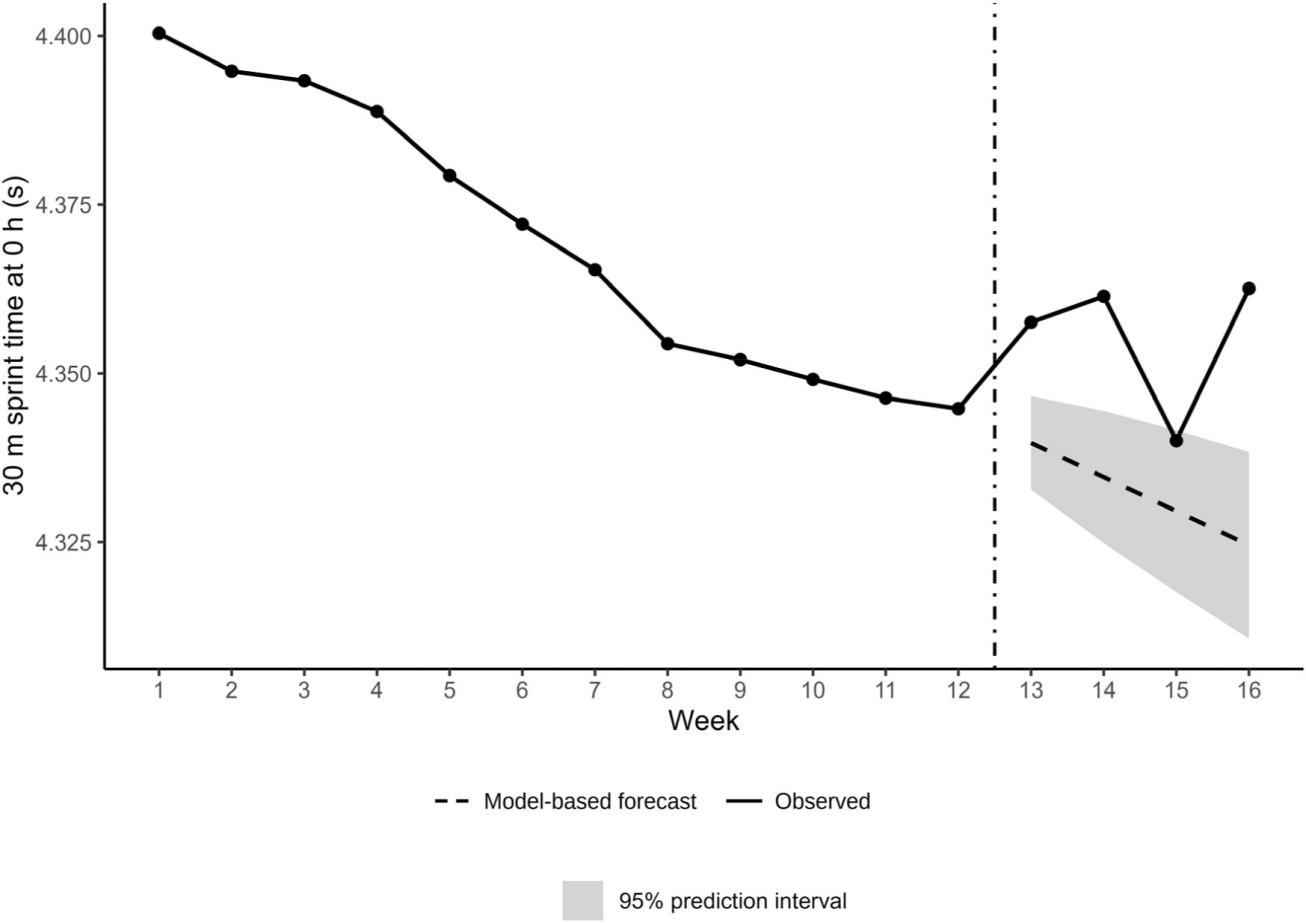
Observed weekly 30 m sprint pre‐session performance and the exploratory weeks 13–16 model‐based forecast with its 95% prediction interval. The vertical dash‐dot line marks the removal of the structured Monday plyometric session. Lower values indicate faster performance.

**FIGURE 4:**
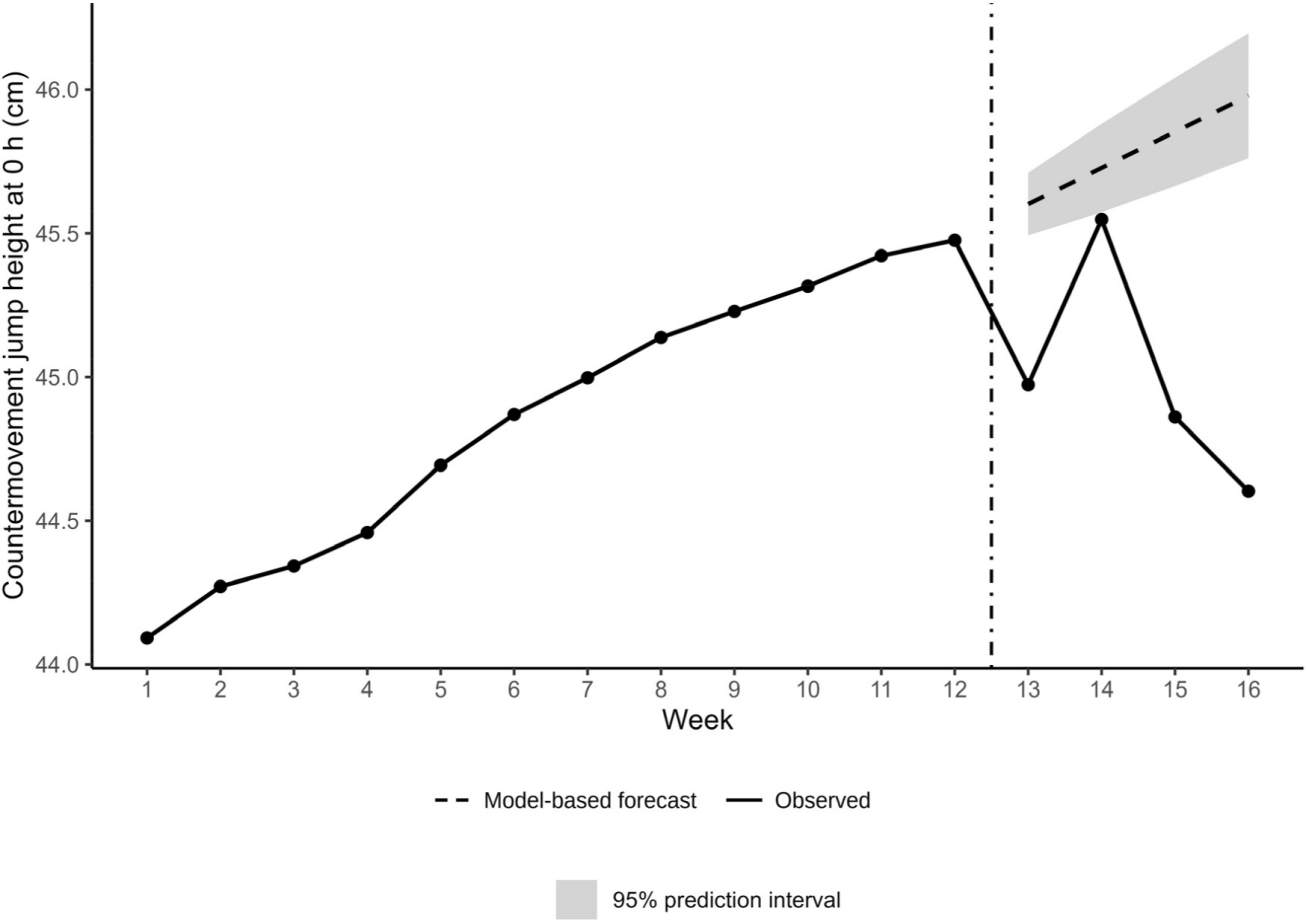
Observed weekly arm‐assisted countermovement jump pre‐session performance and the exploratory weeks 13–16 model‐based forecast with its 95% prediction interval. The vertical dash‐dot line marks the removal of the structured Monday plyometric session. Higher values indicate better performance.

The 72 − 0 h series remained closer: three of four observations for each outcome were within the 95% PI. Week 13 fell outside the favourable direction for both outcomes; weeks 14–16 were within their intervals. Prediction‐interval coverage and MAD values are summarised in Table 7, and the 72 − 0 h trajectories are shown in Figures 5 and 6.

**TABLE 7:** Withdrawal observations relative to exploratory model‐based forecasts.

Series	Observations within 95% PI	MAD
30 m sprint, 0 h	1/4	0.02327 s
CMJ, 0 h	0/4	0.794 cm
30 m sprint, 72 − 0 h	3/4	0.002340 s
CMJ, 72 h − 0 h	3/4	0.079 cm

*Note:* MAD, mean absolute deviation between the observed and forecast values over weeks 13–16; PI, prediction interval. Inclusion within the 95% PI indicates proximity to the model‐based reference and is not a test of equivalence, causation, or physiological retention.

**FIGURE 5:**
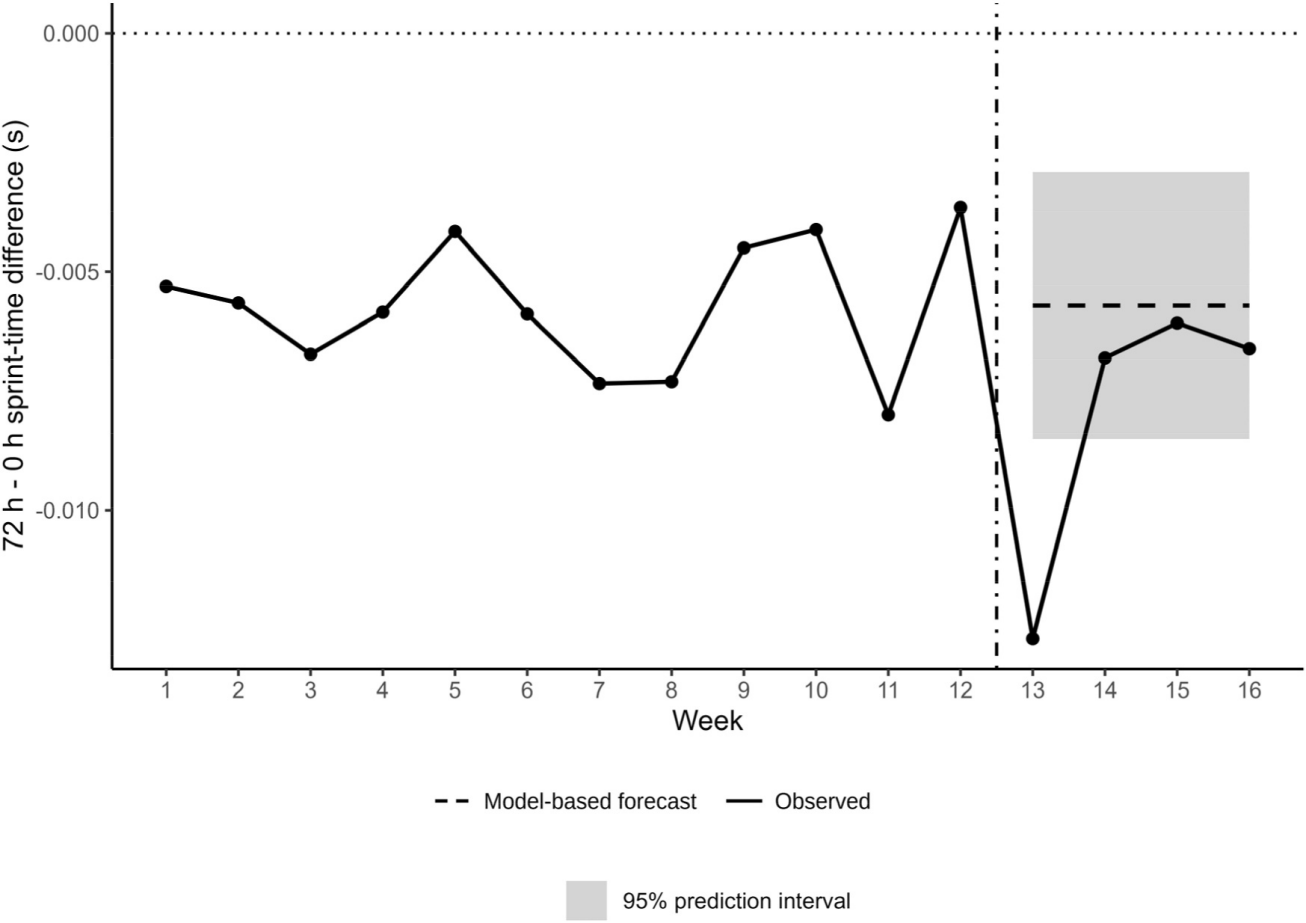
Observed weekly 72 − 0 h difference in sprint time and the exploratory weeks 13–16 model‐based forecast with its 95% prediction interval. The vertical dash‐dot line marks the removal of the structured Monday plyometric session. Negative values indicate faster sprint performance at 72 h than at 0 h.

**FIGURE 6:**
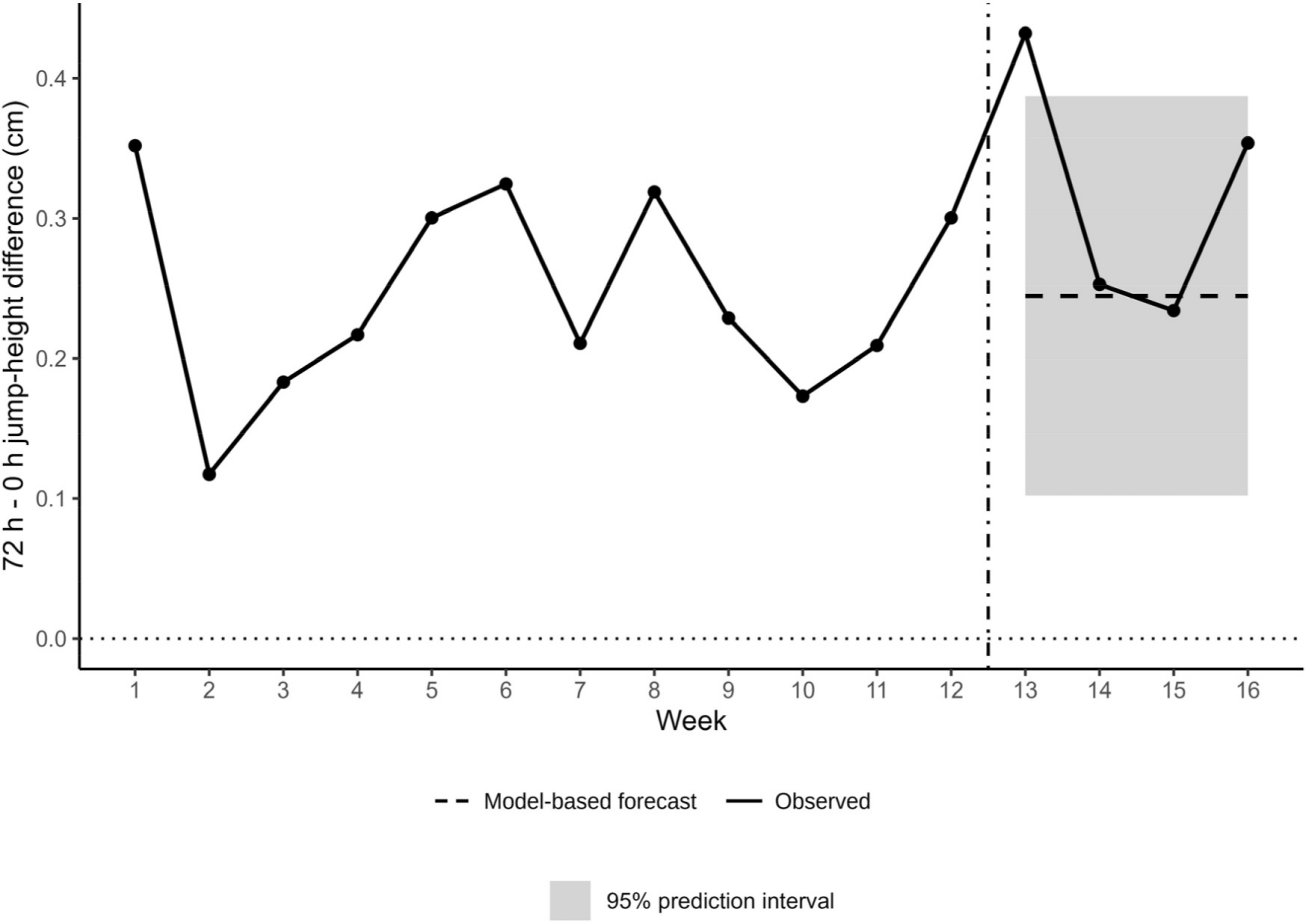
Observed weekly 72 − 0 h difference in arm‐assisted countermovement jump height and the exploratory weeks 13–16 model‐based forecast with its 95% prediction interval. The vertical dash‐dot line marks the removal of the structured Monday plyometric session. Positive values indicate greater jump height at 72 h than at 0 h.

## 4 Discussion

After the structured Monday plyometric session was removed, the weekly pre‐session level and the relative 72 h window did not follow the same course. During the 12 week intervention, sprint time and CMJ height improved, 24 h was generally the least favourable assessment, and 72 h was the most favourable. No week × time‐point interaction was detected, indicating that this within‐week profile did not change systematically as the intervention progressed. The withdrawal results were less uniform. Sprint 0 h performance fell within the model‐based 95% PI in 1 week, whereas CMJ 0 h performance was outside the interval in all 4 weeks. By comparison, three of four 72 − 0 h observations remained within the interval for both outcomes. Thus, the 72 − 0 h contrasts were more often within their respective model‐based intervals than the weekly 0 h values.

The improvement in 0 h performance broadly agrees with earlier plyometric research, particularly the more consistent gains reported for jumping and the generally smaller or more variable changes in sprinting (Markovic 2007; Sáez de Villarreal et al. 2012; Oxfeldt et al. 2019; Kons et al. 2023). The present design does not allow that change to be attributed solely to the Monday programme. The athletes continued their normal sprint curriculum throughout the study, and there was no parallel control group. The finding is therefore best read as an improvement in pre‐session performance while the plyometric block was embedded within the regular sprint schedule. Recent studies also suggest that sprint and jump outcomes do not always respond in parallel, either across development or following training, and should not be treated as interchangeable expressions of one underlying quality (Alexe et al. 2025; Zghal et al. 2025).

The repeated 0–96 h assessments provide a separate view of how performance was expressed within the microcycle. Lower performance at 24 h and better performance at 72 h are consistent with reports that recovery after high‐intensity sprint and jump work may extend over several days (Thomas et al. 2018; Doma et al. 2021). No physiological markers were collected here, so the observed pattern cannot be assigned to muscle damage, neural recovery, tendon behaviour, substrate restoration, or a specific supercompensation process. The result is more limited: under this schedule, average performance was unfavourable at 24 h and favourable at 72 h, and that contrast remained broadly similar across the 12 weeks. CMJ was also slightly higher than 0 h at 96 h, whereas the corresponding sprint contrast was unclear. This may matter when tests or demanding sessions are placed within a similar week, but it does not establish a universal 72 or 96 h optimum.

Interpretation of the withdrawal phase also depends on the training that remained in place. The independent Monday unit was removed, but the athletes continued Friday's sprint training, the standard warm‐up, and a small amount of auxiliary jumping. Sprinting therefore continued to receive a direct task‐specific stimulus, while jump exposure was reduced rather than eliminated. The sprint and CMJ 0 h series differed in their departures from their respective model‐based references, but their MAD values are expressed in different units and cannot establish which adaptation was more durable. The CMJ departures could also reflect differences in the retained training stimulus, the measurement method, or ordinary week‐to‐week variation. Previous detraining studies have likewise shown that speed and power outcomes can diverge when some training continues (Pereira et al. 2020; Nieto‐Acevedo et al. 2025).

The 72 − 0 h contrast adds information that would be missed by tracking the 0 h value alone. In three of the four withdrawal weeks, the 72 − 0 h contrast fell within its model‐based prediction interval for both outcomes, while the 0 h series showed lower coverage overall. Therefore, a coach monitoring only the within‐week contrast might overlook changes in the pre‐session performance level. Monitoring only the 0 h value would miss the opposite possibility. Reporting both measures avoids treating persistence of the relative pattern as evidence that the entire intervention‐related improvement was retained. This distinction is also consistent with the imperfect association between sprint and CMJ performance across populations and test procedures (Oleksy et al. 2024; Zghal et al. 2025).

The exploratory forecasting models were used to extend the intervention‐period series over a short horizon and not to create a true counterfactual. ARIMA produced the lowest rolling errors for all four series, and the Ljung–Box tests did not indicate residual autocorrelation in the final models. Even so, each model was based on only 12 weekly means. The resulting prediction intervals are local and depend on the selected model. They describe how close the withdrawal observations were to the preceding trajectory but cannot show what would actually have occurred had training continued. Both 72 h contrasts were outside the interval in the favourable direction during week 13 and then returned inside it. With only four withdrawal observations, that pattern cannot be confidently separated from normal variation, an immediate response to removal of the Monday session, or other features of the ongoing training week.

The sensitivity analyses were included because the primary random‐intercept models showed heteroscedasticity and serial dependence, particularly for sprint. CR2, CAR(1), and random‐slope models retained the week effect, the principal 24 and 72 h directions, and the absence of a detectable interaction. This gives some support to the main interpretation, although the 48 and 96 h sprint estimates were less stable across error structures. Those comparisons should therefore remain tied to the pre‐specified Tukey‐adjusted results rather than be presented as fully robust across all models. The simulation analysis showed that the realised sample was informative for the stated effect scenarios, but it does not replace prospective sample‐size planning and cannot address the absence of a control group.

## 5 Practical Applications

In programmes organised around a Monday plyometric session and a Friday sprint class, performance assessed on the following day may be less favourable than performance assessed approximately 72 h later. This timing reflects the schedule examined here and should not be treated as a general prescription. During withdrawal or transition phases, coaches may also benefit from monitoring the weekly pre‐session level and the 72 − 0 h difference separately. Persistence of a 72 h advantage does not necessarily mean that the performance level reached during the intervention has been retained.

## 6 Limitations

The study involved one male collegiate sprint squad and did not include a parallel control group. The observed changes therefore cannot be attributed exclusively to the Monday plyometric programme, and their generalisability to female athletes, other age groups, and different sporting populations remains uncertain. The 0 h assessment was obtained within an ongoing sprint programme and represents a weekly pre‐session value rather than an unexposed physiological baseline. Withdrawal removed the independent Monday plyometric session, but the Friday sprint class, standard warm‐up, and a small amount of auxiliary jumping continued. Individual session‐RPE was not collected for the Friday classes, and the contribution of this remaining training to the subsequent 0 h values could not be modelled.

The measurement procedures introduced further constraints. CMJ height was assessed using an arm‐assisted reach‐difference method rather than a force platform, and countermovement depth was self‐selected. Exact hurdle, box, and drop heights, medicine‐ball mass, and the technical specification of the gym surface were not retained in the contemporaneous records. Only the mean of the sprint and CMJ trials was preserved. The original trial values were unavailable, preventing retrospective calculation of the intraclass correlation coefficient, coefficient of variation, or typical error. Equipment, footwear, location, test order, trial number, and recovery intervals were kept consistent, but this procedural control does not replace an independent reliability assessment.

The forecasts were estimated from 12 weekly group means and compared with only four withdrawal observations. Modelling the group mean addressed the applied study question but did not capture individual differences in withdrawal responses. The short intervention series also limits confidence in ARIMA order selection and stationarity testing. Inclusion within a prediction interval should be read as proximity to a model‐based reference, not as evidence of equivalence or physiological retention. No physiological measures were collected, so the 24–96 h pattern describes the timing of performance within this microcycle rather than the mechanism responsible for it.

## 7 Conclusion

Within this cohort and training schedule, 30 m sprint time and arm‐assisted CMJ height improved over the 12 weeks in which a structured Monday plyometric session was included alongside regular sprint training. Performance was less favourable at 24 h and most favourable at 72 h, with no detectable week × time‐point interaction.

After the Monday session was removed, the sprint and CMJ 0 h observations fell within their model‐based 95% prediction intervals in 1/4 and 0/4 weeks, respectively. The corresponding 72 − 0 h observations were within the intervals in 3/4 weeks for both outcomes. The 72 − 0 h contrasts therefore showed higher model‐based interval coverage than the weekly pre‐session measures. These outcomes describe different aspects of short‐term change after withdrawal and should not be treated as interchangeable.

## Author Contributions

All authors contributed equally to the conception, design, data collection, analysis, and writing of the manuscript. All authors have read and approved the final manuscript for publication.

## Funding

The authors have nothing to report.

## Conflicts of Interest

The authors declare no conflicts of interest.

## Supporting information


Supporting Information S1

## Data Availability

The data that support the findings of this study are available from the corresponding author upon reasonable request.
