# Supplementary material for: Baseline Performance and the 72‐Hour Performance Window Following Plyometric Withdrawal in Collegiate Sprinters
Source: Eur J Sport Sci. 2026 Oct 20;26(11):e70277. doi: 10.1002/ejsc.70277 (PMC13683558; doi:10.1002/ejsc.70277)
Supplement: Supplementary file 1 [file EJSC-26-e70277-s001.docx]

**Supporting Information**

**Baseline Performance and the 72-Hour Performance Window Following Plyometric Withdrawal in Collegiate Sprinters**

# **Supplementary Table S1. Structured Monday plyometric programme**

| **Week** | **Phase** | **Exercises** | **Sets** | **Repetitions per set** | **Jump contacts** | **Between sets** | **Between exercises** |
| --- | --- | --- | --- | --- | --- | --- | --- |
| 1 | Phase 1 | Unilateral vertical hurdle jumps; bilateral vertical hurdle jumps; unilateral lateral hurdle jumps; bilateral lateral hurdle jumps; unilateral zig-zag hurdle jumps; bilateral zig-zag hurdle jumps | 3 per exercise | 8 per set | 144 | 1 min | 3 min |
| 2 | Phase 1 | Unilateral vertical hurdle jumps; bilateral vertical hurdle jumps; unilateral lateral hurdle jumps; bilateral lateral hurdle jumps; unilateral zig-zag hurdle jumps; bilateral zig-zag hurdle jumps | 3 per exercise | 8 per set | 144 | 1 min | 3 min |
| 3 | Phase 1 | Unilateral vertical hurdle jumps; bilateral vertical hurdle jumps; unilateral lateral hurdle jumps; bilateral lateral hurdle jumps; unilateral zig-zag hurdle jumps; bilateral zig-zag hurdle jumps | 3 per exercise | 10 per set | 180 | 1 min | 4 min |
| 4 | Phase 1 | Unilateral vertical hurdle jumps; bilateral vertical hurdle jumps; unilateral lateral hurdle jumps; bilateral lateral hurdle jumps; unilateral zig-zag hurdle jumps; bilateral zig-zag hurdle jumps | 3 per exercise | 12 per set | 216 | 1 min | 4 min |
| 5 | Phase 2 | Box jumps; depth jump to unilateral hurdle jump; lateral push-off to box jump; overhead medicine-ball throw; backward medicine-ball throw | 3 per exercise | 8; 8; 10; 10; 10 | 78 | 1 min | 3 min |
| 6 | Phase 2 | Box jumps; depth jump to unilateral hurdle jump; lateral push-off to box jump; overhead medicine-ball throw; backward medicine-ball throw | 3 per exercise | 8; 8; 10; 10; 10 | 78 | 1 min | 3 min |
| 7 | Phase 2 | Box jumps; depth jump to unilateral hurdle jump; lateral push-off to box jump; overhead medicine-ball throw; backward medicine-ball throw | 3 per exercise | 10; 10; 15; 15; 15 | 105 | 1 min | 4 min |
| 8 | Phase 2 | Box jumps; depth jump to unilateral hurdle jump; lateral push-off to box jump; overhead medicine-ball throw; backward medicine-ball throw | 3 per exercise | 12; 12; 20; 20; 20 | 132 | 1 min | 4 min |
| 9 | Phase 3 | Depth jump to box; unilateral tuck jump; half-squat to vertical box jump; cyclical split-squat jump; split-scissor jump | 3 per exercise | 8; 8; 8; 10; 10 | 132 | 1 min | 3 min |
| 10 | Phase 3 | Depth jump to box; unilateral tuck jump; half-squat to vertical box jump; cyclical split-squat jump; split-scissor jump | 3 per exercise | 8; 8; 8; 10; 10 | 132 | 1 min | 3 min |
| 11 | Phase 3 | Depth jump to box; unilateral tuck jump; half-squat to vertical box jump; cyclical split-squat jump; split-scissor jump | 3 per exercise | 10; 10; 10; 12; 12 | 162 | 1 min | 4 min |
| 12 | Phase 3 | Depth jump to box; unilateral tuck jump; half-squat to vertical box jump; cyclical split-squat jump; split-scissor jump | 3 per exercise | 12; 12; 12; 15; 15 | 198 | 1 min | 4 min |

*Note. All sessions were held Monday 13:30–15:00 and were completed by all 26 athletes. Medicine-ball throws were not included in jump-contact totals. For unilateral exercises, set 1 used the left leg, set 2 the right leg, and set 3 the preferred force-production leg. Exact hurdle, box, and drop heights, medicine-ball mass, and the gym-surface specification were not retained in the contemporaneous record and were not reconstructed retrospectively.*

# **Supplementary Table S2. Standardised warm-up and test procedures**

| **Component** | **Dose** | **Content** | **Control** |
| --- | --- | --- | --- |
| Stationary preparation | 4 min | Head movements; shoulder circles; chest opening and arm swings; trunk rotation and hip circles; forward and lateral lunges; low-amplitude ankle bouncing | Controlled range of motion, gradually increased |
| Ankle jump–A-jump–acceleration | 2 sets | 10 m ankle jumps + 10 m A-jumps + 20 m progressive acceleration | Approximately 70% at the end |
| Alternate bound–high-knee run–acceleration | 2 sets | 10 m low-amplitude alternate bounds + 10 m high-knee run + 20 m acceleration | Approximately 75–80% at the end |
| Single-leg exchange–short-stride run–acceleration | 1–2 sets | 10 m low-amplitude alternating exchanges + 10 m rapid short-stride run + 20 m acceleration | Approximately 80–85% at the end |
| Event-specific progressive run | 1 trial | 40–50 m progressive run; straight for 100 m athletes and curve permitted for 200 m athletes | Approximately 85–90%; no all-out sprint |

*Note. The same 10 min warm-up was used before all 0–96 h assessments, withdrawal assessments, Monday plyometric sessions, and Friday sprint classes.*

# **Supplementary Table S2 (continued). Performance-test procedures**

| **Test** | **Location** | **Instrument** | **Footwear** | **Trials** | **Rest** | **Recorded outcome** |
| --- | --- | --- | --- | --- | --- | --- |
| CMJ | Gym; same flat white wall | Mijia MJJGCJY001QW laser distance meter | Ordinary running shoes | 3 | ≥3 min | Arithmetic mean of three reach-difference trials |
| 30 m sprint | Adjacent indoor track; athlete-specific fixed lane | CC01 single-point infrared timing system | Athlete-specific fixed sprint spikes | 2 | ≥10 min | Arithmetic mean of two electronically timed trials |

*Note. CMJ was arm-assisted, used self-selected countermovement depth, and was calculated as jump reach minus standing reach. Sprint timing began and ended when the athlete broke the respective infrared beams. The trial means were retained; individual trial records were not preserved.*

# **Supplementary Table S3. Adherence, safety, and withdrawal-phase exposure**

| **Item** | **Record** |
| --- | --- |
| Participants enrolled | 26/26 of the eligible available squad |
| Structured Monday plyometric sessions | 312/312 participant-sessions completed |
| Planned performance assessments | 1,768/1,768 participant-assessment occasions completed |
| Core missing outcome values | 0 |
| Study withdrawals | 0 |
| Protocol modifications | 0 |
| Mild transient adverse events | 3 athletes: two brief dizziness episodes and one brief nausea episode; resolved after 3–4 min rest; no medication, referral, or withdrawal |
| Weeks 13–16 Monday exposure | No independent structured plyometric session |
| Weeks 13–16 Friday exposure | One regular sprint-specific class per week, 16:00–17:30; all 26 completed |
| Other withdrawal exposure | Standard warm-up and small auxiliary jumping within regular teaching continued; no replacement plyometric, separate resistance, or additional explosive programme |
| Friday/withdrawal individual load records | Not retained as individual session-RPE data |

# **Supplementary Table S4. Model diagnostics and sensitivity analyses**

| **Outcome** | **Check/model** | **Result** | **Interpretation** |
| --- | --- | --- | --- |
| Sprint | Shapiro–Wilk | W = 0.9960; p = .00041 | Small formal departure; Q–Q deviation concentrated in the tails |
| CMJ | Shapiro–Wilk | W = 0.9988; p = .350 | No clear departure |
| Sprint | Breusch–Pagan | χ² = 9.582; p = .00196 | Heteroscedasticity indicated |
| CMJ | Breusch–Pagan | χ² = 6.961; p = .00833 | Heteroscedasticity indicated |
| Sprint | Mean within-athlete lag-1 residual correlation | .741 | Serial dependence present |
| CMJ | Mean within-athlete lag-1 residual correlation | .319 | Moderate serial dependence |
| Sprint | Continuous-time CAR(1) | .865 per day | Core fixed-effect directions retained |
| CMJ | Continuous-time CAR(1) | .334 per day | Core fixed-effect directions retained |
| Sprint and CMJ | Participant random week slope | Both models non-singular | Week effect and non-significant interaction retained |
| Sprint and CMJ | Participant-clustered CR2 | Core week, 24 h, and 72 h conclusions retained | Used as a sensitivity analysis, not as replacement pairwise inference |

# **Supplementary Table S4 (continued). Simulation-based sensitivity scenarios**

| **Scenario** | **Outcome** | **Coefficient** | **Assumed effect** | **Meaning** | **Power** | **95% CI** |
| --- | --- | --- | --- | --- | --- | --- |
| 1 | Sprint | week_c | -0.001 | weekly change in 0 h sprint time (s/week) | 0.960 | [0.950, 0.968] |
| 4 | Sprint | timepoint72 | -0.002 | 72 h minus 0 h sprint-time contrast at centered week (s) | 0.323 | [0.302, 0.343] |
| 5 | Sprint | timepoint72 | -0.004 | 72 h minus 0 h sprint-time contrast at centered week (s) | 0.869 | [0.853, 0.883] |
| 6 | Sprint | timepoint72 | -0.006 | 72 h minus 0 h sprint-time contrast at centered week (s) | 0.995 | [0.991, 0.997] |
| 7 | CMJ | week_c | 0.03 | weekly change in 0 h CMJ height (cm/week) | 1.000 | [0.997, 1.000] |
| 10 | CMJ | timepoint72 | 0.1 | 72 h minus 0 h CMJ contrast at centered week (cm) | 0.944 | [0.933, 0.953] |
| 11 | CMJ | timepoint72 | 0.2 | 72 h minus 0 h CMJ contrast at centered week (cm) | 1.000 | [0.998, 1.000] |
| 12 | CMJ | timepoint72 | 0.3 | 72 h minus 0 h CMJ contrast at centered week (cm) | 1.000 | [0.998, 1.000] |

*Note. Each scenario used 2,000 simulations and α = .05. These analyses describe selected assumed effects under the realised N = 26 repeated-measures design; they were not prospective sample-size calculations and were not used to infer power from the observed effects.*

# **Supplementary Table S5. Regular Friday sprint training maintained across weeks 1–16**

| **Week** | **Focus** | **Main running content** | **Planned total/high-intensity volume** |
| --- | --- | --- | --- |
| 1 | Technical calibration and acceleration | 4 × 20 m three-point starts; 4 × 30 m block starts; 3 × 60 m acceleration-extension runs | 560 m total; 380 m high-intensity |
| 2 | Technical calibration and acceleration | 4 sets of resisted 20 m + unresisted 30 m; 3 × 50 m acceleration-extension runs | 570 m total; 350 m high-intensity |
| 3 | Transition to maximum velocity | 4 × (30 m build-up + 20 m flying); 3 × 60 m block starts; 2 × 80 m relaxed fast runs | 720 m total; 540 m high-intensity |
| 4 | Reduced-load technical review | 4 × 30 m block starts; 3 × 60 m block starts; 1 × 80 m rhythm run | 560 m total; 380 m high-intensity |
| 5 | Maximum velocity | 4 × (20 m build-up + 30 m flying); 3 × 60 m variable-speed runs; 2 × 80 m speed-maintenance runs | 720 m total; 540 m high-intensity |
| 6 | 100 m speed endurance | 3 × 80 m; 2 × 120 m | 660 m total; 480 m high-intensity |
| 7 | 200 m curve and transition | 4 × 60 m curve starts; 3 × 120 m curve-to-straight runs | 780 m total; 600 m high-intensity |
| 8 | Speed endurance | 2 × 150 m; 2 × 120 m | 720 m total; 540 m high-intensity |
| 9 | 100 m race model | 4 × 30 m block starts; 3 × 60 m block starts; 2 × 100 m model runs | 680 m total; 500 m high-intensity |
| 10 | 200 m specific endurance | 2 × 180 m; 1 × 150 m | 690 m total; 510 m high-intensity |
| 11 | Segment combination | 2 sets of 120 m + 80 m (60 s within-set recovery); 2 × 60 m relaxed fast runs | 700 m total; 520 m high-intensity |
| 12 | 100/200 m model review | 3 × 30 m starts; 2 × 60 m; 100 m group: 1 × 80 m + 1 × 100 m; 200 m group: 2 × 90 m | 570 m total; 390 m high-intensity |
| 13 | Withdrawal-phase speed maintenance | 4 × (30 m build-up + 20 m flying); 2 × 60 m starts; 1 × 120 m | 620 m total; 440 m high-intensity |
| 14 | Withdrawal-phase speed endurance | 2 × 150 m; 2 × 80 m | 640 m total; 460 m high-intensity |
| 15 | Pre-assessment taper | 3 × 30 m starts; 2 × 60 m; 100 m group: 1 × 120 m; 200 m group: 1 × 120 m + 1 × 80 m | 100 m: 510/330 m; 200 m: 590/410 m |
| 16 | Event assessment | 2 × 30 m timed starts; 1 × 60 m rehearsal; 1 × 100 m or 1 × 200 m timed effort | 100 m: 400/220 m; 200 m: 500/320 m |

*Note. The Friday class was held from 16:00 to 17:30. Planned volumes were taken from the final course record; values before and after the slash in weeks 15–16 are total and high-intensity running volumes, respectively. The standard 10 min study warm-up and a small amount of auxiliary jumping continued, but the latter was not separately quantified and did not constitute a replacement plyometric session.*
